# High Sensitivity C-Reactive Protein as a Prognostic Indicator of Cardiovascular Disease in Severe Non-Diabetic COVID-19 Patients

**DOI:** 10.15190/d.2023.11

**Published:** 2023-09-18

**Authors:** Mugundhan Kandhasami, Subash Panchanathan, Jayanthi Rajendran, Karthik Balajee Laksham

**Affiliations:** ^1^Department of Biochemistry, Jawaharlal Institute of Postgraduate Medical Education and Research (JIPMER), Karaikal-609602, Puducherry, India; ^2^Department of Biochemistry, Mahatma Gandhi Medical College and Research Institute, "Sri Balaji Vidyapeeth (Deemed -to be-University)," Puducherry-607402; ^3^Department of Community Medicine, Jawaharlal Institute of Postgraduate Medical Education and Research (JIPMER), Karaikal-609602, Puducherry, India

**Keywords:** Hyperglycemia, hs-CRP, Cardiovascular disease, Non-Diabetic, COVID-19 ICU patients.

## Abstract

OBJECTIVE: The long-term extrapulmonary sequelae of COVID-19 after recovery from the critical stage at the intensive care unit (ICU) are still unclear. Some post-COVID symptoms are prevalent even after a one-year follow-up. To explore the relationship between high sensitivity C-reactive protein (hs-CRP) and hyperglycemia with cardiovascular diseases in non-diabetic COVID-19 patients. To determine whether increased fasting blood sugar (FBS) levels are associated with elevated hs-CRP and to explore whether hs-CRP can serve as a prognostic indicator to predict cardiovascular outcome. METHODS: FBS and hs-CRP values of 26 non-diabetic COVID-19 patients were collected from their medical records at JIPMER hospital. In one-year follow-up of these 26 patients, 2mL of blood sample was collected for the analysis of FBS, HbA1c, and hs-CRP. RESULTS: hs-CRP increased in 23% of follow-up patients who were at high risk, and 42.3% of participants were at average risk for cardiovascular disease. High and average-risk groups of survivors showed a positive correlation of hs-CRP with FBS and HbA1c levels, and these patients should be carefully monitored. CONCLUSION: ICU survivors with elevated hs-CRP need periodic check-ups for cardiovascular diseases. We suggest that hs-CRP could be used as an early prognostic indicator of cardiovascular diseases and can reduce the risk.

## INTRODUCTION

As of June 23, 2023, the World Health Organization reported over 634 million global cases of Coronavirus disease 2019 (COVID-19), with more than 6.8 million deaths and almost 663 million recoveries worldwide^1^. The long-term complications of COVID-19 are a major concern, particularly among critically ill patients who received intensive care treatment. Multiple deleterious cardiovascular effects are associated with the coronavirus, including damage to the heart muscle and impaired cardiac function^2,3^. Early studies have shown an increased incidence of major cardiovascular events after hospitalization^4^, highlighting the association between COVID-19 and cardiovascular disease^5^.

Several studies have documented the association between COVID-19 severity and inflammatory and coagulation makers such as D-dimer, prothrombin time (PT) and C-reactive protein (CRP) as indicator of inflammation disorder and coagulopathy respectively^6,7^. Cardiac markers like Troponin I, creatine kinase-myoglobin binding (CK-MB), and myoglobin are measured to evaluate cardiac function. However, there are no early markers for predicting disease severity and progression in COVID-19 survivors to prevent increased mortality. As hs-CRP is a sensitive marker of inflammation, high levels of hs-CRP are linked to an increased risk of future heart attack, stroke, cardiac failure, peripheral arteries diseases and cardiac events^8^and also economically cheaper compared to other cardiac markers.

The hs-CRP increases the recruitment of monocytes into atheromatous plaque and also inducing endothelial dysfunction by suppressing basal and induced nitric oxide release^9^. Additionally, it has been observed that hs-CRP alters macrophage absorption of low-density lipoprotein (LDL) and upregulates the expression of vascular endothelial plasminogen activator inhibitor-1 and other adhesion molecules^10^. Hence in this study we assessed the prognostic value of high sensitivity C-reactive protein (hs-CRP) for predicting cardiovascular diseases (CVD) risk in COVID-19 survivors.

COVID-19 virus stimulates gluconeogenesis that results in hyperglycemia which induces accumulation of reactive oxygen species (ROS)^11^.

This ROS leads to irreversible oxidative modifications causing endothelial dysfunction which plays a central role in the pathogenesis of micro- and macro-vascular diseases. ROS also increase the expression of pro-inflammatory factors, inducing atherosclerotic lesions and vascular smooth-muscle cell apoptosis, leading to atherosclerotic plaque instability and rupture^12^. Acute hyperglycemia in ICU survivors is more dangerous for people without diabetes than those with diabetes^13^. Furthermore, there is a strong association between fasting plasma glucose level and hs-CRP^14^.

The aim of this study is to evaluate the prognostic value of hs-CRP levels at Admission, Discharge, and one-year follow-up in predicting cardiovascular risk among non-diabetic COVID-19 survivors at JIPMER, Puducherry, India.

The results of this study could provide important insights into the long-term cardiovascular risks associated with COVID-19, identify the people who will become critically ill, and contribute to the development of effective risk prediction and management strategies for COVID-19 survivors.

## MATERIALS AND METHODS

### Population and Sample

Our study is a longitudinal, single-centre clinical follow-up investigation that aims to explore the clinical course of COVID-19 patients admitted to the intensive care unit (ICU) of JIPMER, Puducherry, between April 2021 and September 2021. Both males and females in the age group of 30 to 75 years who tested positive for SARS-CoV-2 by PCR (polymerase chain reaction) and exhibited typical COVID-19 symptoms according to the World Health Organization (WHO) guidelines^15^ were included in the study in accordance with the diagnostic criteria. Non-Diabetic patients, either with normoglycemic or new-onset hyperglycemia diagnosed with COVID-19 positive during the time of ICU admission. Patients with a history of pre-existing underlying chronic systemic diseases such as tuberculosis, asthma, autoimmune diseases, diabetes mellitus, hypertension, thyroid illness, and other chronic organ dysfunction that may present with signs and symptoms as post-COVID-19 symptoms were excluded from this study.

### Data Collection

Baseline demographic, clinical, laboratory, radiological, treatment, and outcomes data were collected from in-hospital electronic medical records of each patient. Patients' long-term outcome data were obtained via mobile phone after hospital discharge. A post-COVID-19 camp was arranged for blood sample collection one year later.

### Blood Sample Collection

2mL of fasting blood was collected and centrifuged at 3000 rpm for 10 minutes for serum separation. Serum sample was used for the analysis of fasting blood glucose by the Glucose oxidase peroxidase method, hs-CRP by ELISA kit (Enzyme-Linked Immunosorbent Assay) from Diagnostics Biochem in Canada, and HbA1c by high-performance liquid chromatographic (HPLC) method using reagent kits from Biorad (D10). Normal FBS is 70-100 mg/dL, FBS between 101-125 mg/dL is considered as impaired glucose /pre-diabetic, FBS >126 mg/dL is considered as Diabetic.

### Statistical analysis

Statistical Analysis was done using R software ‘Psych’ and ‘ggpubr' packages. Data were expressed as means ± standard deviation. Boxplot shows FBS and hs-CRP levels at different time intervals. A scatter plot was used to find the correlation between FBS and hs-CRP levels during admission at ICU and after a one-year follow-up. Pearson correlation was done, and 'r' value was determined. The chi-square test would be used to assess the differences in the continuous and categorical variables, respectively. *P *value < 0.05 was considered statistically significant.

### Ethical Considerations

The study was approved by Institutional Ethics Committee (JIP/IEC-OS/2022/252). Written informed consent was provided to all the participants prior to inclusion. A patient information sheet (PIS) and questionnaire were taken.

## RESULTS

**Figure 1:**
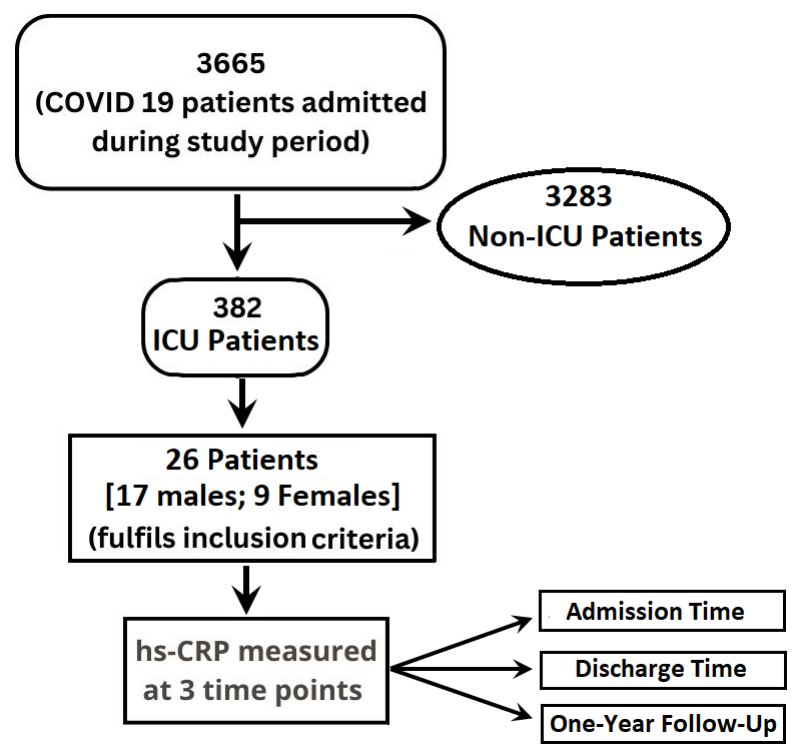
Flowchart representation of COVID-19 ICU patients

In the study period, 382 critically ill patients were admitted to ICU, JIPMER-Puducherry. Twenty-six patients met the inclusion criteria, 9 (34.6%) were women and 17 (65.4%) were men as shown in study design of COVID-19 ICU patients (Figure 1). The participants' mean (SD) age is 52.4 (13.6) years. The median (range) duration of their hospital stay is 16.5 (7-118) days and the levels of fasting blood sugar and hs-CRP is expressed in mg/dL and mg/L respectively (Table 1).

**Table 1:** The demographic and clinical characteristics of the COVID-19 ICU patients at different time points, n=26

S. No	Characteristic	Mean (SD)	Median	Minimum	Maximum
1	Age (Years)	52.4 (13.6)	52	30	75
2	Hospital Stay duration (Days)	27.1 (25.6)	16.5	7	118
3	FBS (mg/dL)				
	Admission	160.3 (44.8)	164	98	283
	Discharge	109.3 (25.1)	108.5	75	205
	One Year	95.3 (11.7)	92	80	130
4	hs-CRP (mg/L)				
	Admission	37.2 (20.2)	32.8	10.4	72
	Discharge	12.5(8.5)	11.4	2.9	28
	One Year	2.1(2.0)	1.5	0.2	7

**Figure 2:**
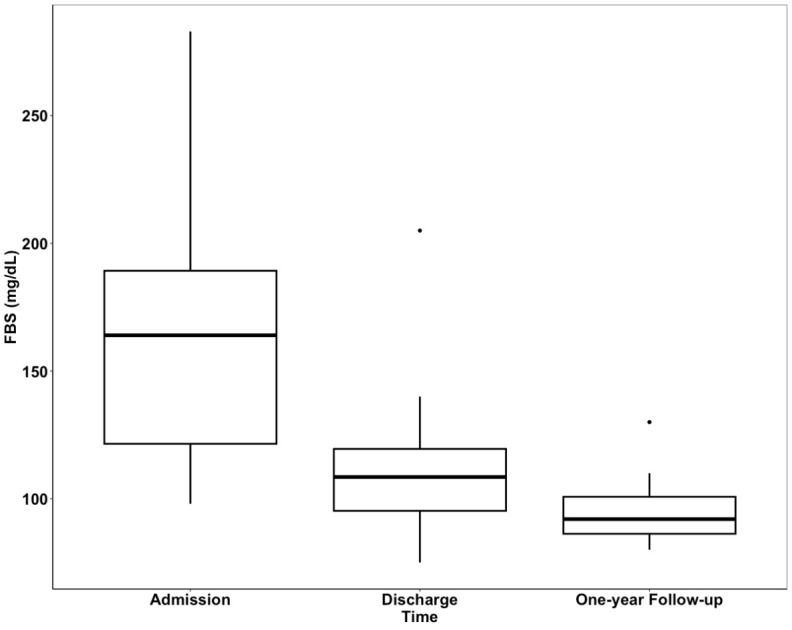
Boxplot showing FBS levels at different time intervals FBS, fasting blood sugar.

The Fasting Blood Sugar is categorized as Normal, and hyperglycaemia using 126 mg % as cut off. The mean (SD) FBS levels (mg/dL) of the study participants at different time intervals are 160.3 (44.8) at Admission, 109.3 (25.1) at Discharge, and 95.3 (11.7) at One year, and the distribution of Fasting Blood Sugar (Figure 2).

Only three participants had FBS above 126 mg% at the time of Discharge. The number of patients with hyperglycemia decreased from Admission to one-year follow-up; this trend is statistically significant (p<0.001) (Table 2).

**Table 2:** Glycemic status of non-diabetic COVID-19 ICU patients at different time points

Glycemic status	Admission (n=26)	Discharge (n=26)	One Year (n=26)	Statistics
Normo-glycemic	7	23	25	Chi-square trend test = 30.0, p < 0.001
Hyper-glycemic	19	3	1	

**Figure 3:**
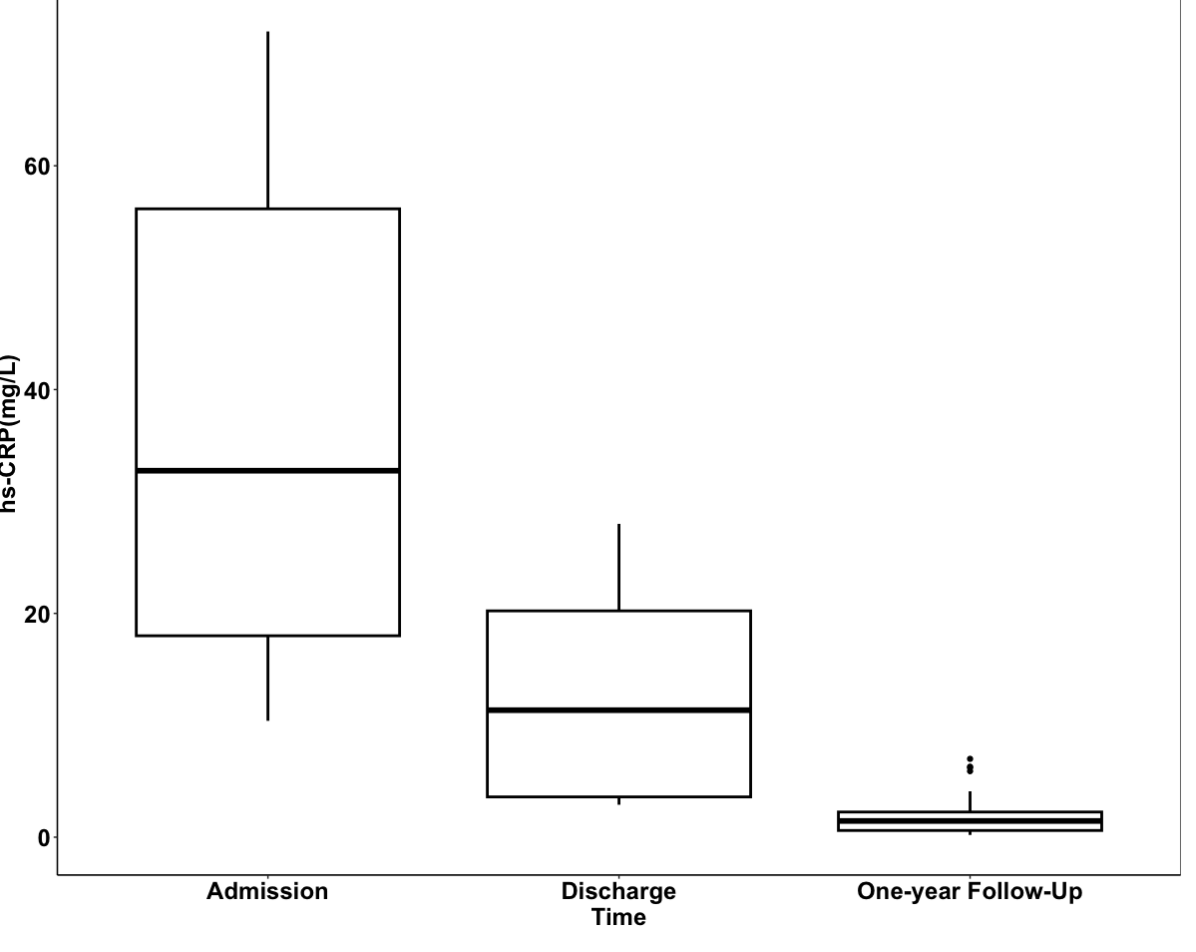
Boxplot showing hs-CRP levels at different time intervals hs-CRP, high sensitivity C-reactive protein.

The mean (SD) hs-CRP levels (mg/L) at different time intervals are 37.2 (20.2) at Admission, 12.5 (8.5) at Discharge, and 2.1 (2.0) at one-year follow-up. The distribution of hs-CRP levels at different time intervals (Figure 3).

The hs-CRP levels are categorized as Normal (<1 mg/L), Average Risk (1-3 mg/L), and High Risk (>3 mg/L). All patients had a High-Risk level of hs-CRP during Admission, which decreased during 1-year follow-up. The declining trend in s-CRP levels is statistically significant (p<0.001) (Table 3).

**Table 3:** hs-CRP status of COVID-19 ICU patients at different time points hs-CRP, high sensitivity C-reactive protein

hs-CRP category	Admission (n=26)	Discharge (n=26)	One Year (n=26)	Statistic
Normal	0	0	9	Chi-square trend test = 30.0, p < 0.001
Average Risk	0	4	11	
High Risk	26	22	6	

**Figure 4:**
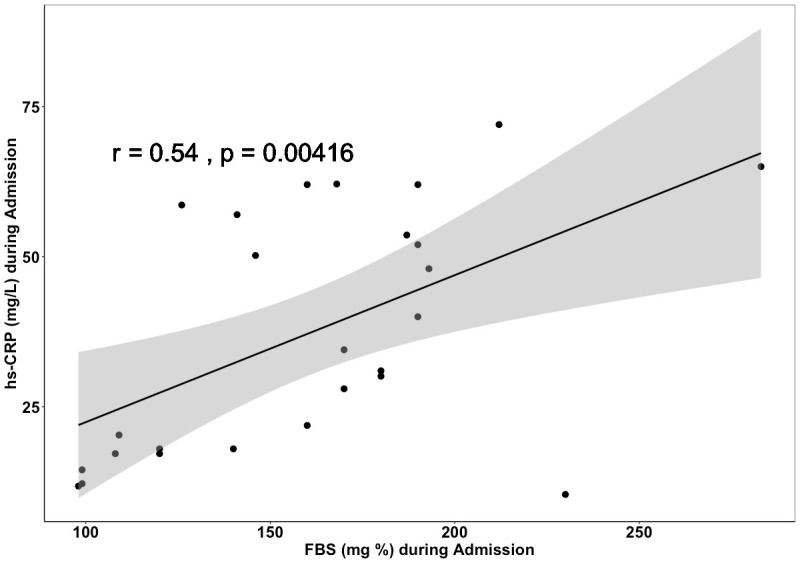
Correlation between FBS and hs-CRP during admission FBS, fasting blood sugar; hs-CRP, high sensitivity C-reactive protein.

**Figure 5:**
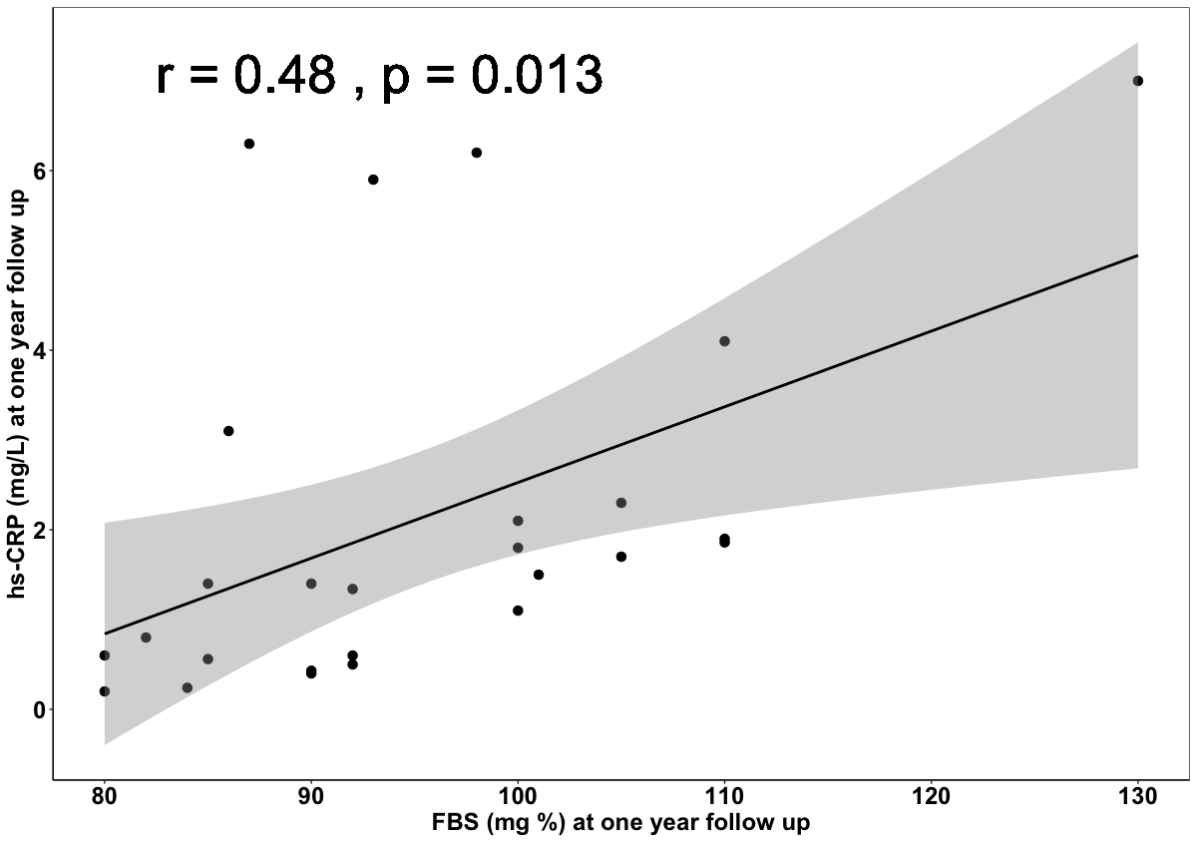
Correlation between FBS and hs-CRP during one-year follow-up FBS, fasting blood sugar; hs-CRP, high sensitivity C-reactive protein.

There is a positive correlation (r=0.54, *p*=0.004) between the level of fasting blood sugar and hs-CRP during admission (Figure 4) and after one-year of discharge from the hospital, positive correlation (r=0.48, *p*=0.013) (Figure 5). The mean (SD) of HbA1C levels after a one-year follow-up is 6.7 (1.0), ranging from 5.2 to 9.1.

## DISCUSSION

Our study reports one-year follow-up data of patients with severe COVID-19 who were discharged from the hospital ICU. Most patients reported that their quality of life was altered after rehabilitation from COVID-19. Weariness had become a common symptom, leading to limitations in physical mobility and, in a few cases, difficulty carrying out their previous job. Several studies have suggested that SARS-CoV-2 patients may develop hyperglycemia that promotes viral replication and, thereby, increases viral load^16,17^. Also, hyperglycemia promotes the synthesis of AGEs (advanced glycation end products), reactive oxygen species (ROS), and proinflammatory cytokines^18^. Elevated glucose levels suppress the antiviral immune response, cause pulmonary dysfunction, and leads to fatal outcomes^19,20^. Even without a previous history of diabetes mellitus, the virus has been shown to cause the failure of the islets of Langerhans and activation of dipeptidyl peptidase 4 (DPP4), thus inhibiting insulin secretion and provoking hyperglycemia^21^. Thus, our findings add a reliable association between SARS-CoV-2 and hyperglycemia.

Elevated CRP levels have been correlated with disease severity and serve as a highly sensitive biomarker for inflammation^22^. In the present study, serum hs-CRP levels were measured instead of CRP, as high levels of hs-CRP in the blood are closely linked to increased cardiovascular risk^23^. The results of the present study reveal that some ICU-COVID patients, even after a one-year follow- up, are at risk of CVD complications due to the higher inflammation and extreme stress they challenged during their treatment period.

As recommended by the American Heart Association^24^, patients at intermediate or high risk of coronary heart disease may benefit from the measurement of hs-CRP and future cardiovascular risk. Positive results of this study will further strengthen hs-CRP as a prognostic marker for cardiovascular disease risk in COVID-19 follow-up patients.

The study found that very high values of hs-CRP in group-1 indicated an inflammatory response due to the critical illness of COVID-19 infection in the ICU. A statistically significant decrease in hs-CRP levels was observed between group-1 and 2 and also between group-1 and 3, indicating better responses of critical COVID-19 patients to their effective treatment. However, the study also revealed that hs-CRP levels of 23% of follow-up ICU survivors were above 3mg/L who were at high risk for CVD, and 42.3% of patients had hs-CRP between 1-3mg/L with average risk.

Additionally, only 3.8% of diabetic COVID-19 ICU patients showed high HbA1c after one year follow-up. High HbA1c levels are a risk factor for severity^25^. Therefore, it is important to measure HbA1c in COVID-19 ICU patients.

### Benefits and Limitations

The current study encompasses a short sample size. Furthermore, clinical studies with larger sample sizes may be needed. Levels of hs-CRP compared with multiple cardiac markers are warranted to establish complete long-term complications of post-COVID-19 that can be compared with our current findings. Also, HbA1c was not performed during their hospital stay at ICU. Furthermore, information about post-ICU treatment, such as rehabilitation program, was unavailable. This information could have been valuable to better interpret one-year outcomes.

## CONCLUSION

Our finding shows elevated hs-CRP levels in approximately one-fourth of the ICU survivors. We suggest that serum hs-CRP could be used as an essential early prognostic indicator of cardiovascular disease. More critical COVID-19 ICU patients should be carefully monitored even after discharge.

Furthermore, checking hs-CRP levels once in a while for better monitoring, managing, and predicting CVD to reduce the risk may be planned accordingly.
